# SDCBP2 promotes tumor progression and is a novel ferroptosis-related prognostic biomarker in lung adenocarcinoma

**DOI:** 10.3389/fimmu.2025.1692308

**Published:** 2025-12-02

**Authors:** Shaowu Sun, Zhuoyu Gu, Shuang Yuan, Kaishang Zhang, Wei Liu, Zhengpan Wei, Wenbo Fan, Zelong Wang, Tianliang Zhao, Mingbo Tang, Xinliang Gao, Guoqing Zhang, Xiangnan Li, Xue Pan

**Affiliations:** 1 State Key Laboratory of Metabolic Dysregulation & Prevention and Treatment of Esophageal Cancer, The First Affiliated Hospital of Zhengzhou University, Zhengzhou University, Zhengzhou, Henan, China; 2 Department of Thoracic Surgery, The First Affiliated Hospital of Zhengzhou University, Zhengzhou University, Zhengzhou, Henan, China; 3 Department of Thoracic Surgery, The First Hospital of Jilin University, Changchun, Jilin, China; 4 Henan Medical Key Laboratory of Thoracic Oncology, Zhengzhou, Henan, China; 5 Henan Molecular Pathology and Clinical Experimental Engineering Research Center of Thoracic Disease, Zhengzhou, Henan, China; 6 Nursing College of Zhengzhou University, Zhengzhou University, Zhengzhou, Henan, China

**Keywords:** lung adenocarcinoma, SDCBP2, prognostic marker, ferroptosis, cell cycle, apoptosis

## Abstract

**Introduction:**

Lung adenocarcinoma (LUAD) is a global health threat due to its rapid malignant progression and poor prognosis. Ferroptosis plays a key role in LAUD progression, but the critical ferroptosis-related factors in LUAD have not been further explored. The aim of this work was to explore novel ferroptosis-related therapeutic targets and prognostic biomarkers for LUAD.

**Methods:**

Using TCGA and GEO datasets of LAUD samples, we constructed a ferroptosis risk model and identified SDCBP2 as a LUAD hub gene. The TCGA data and single-cell database indicated that SDCBP2 was overexpressed in LUAD and significantly enriched in malignant cells. Subsequently, bioinformatics analysis, functional studies, and clinical samples were employed to assess the prognostic value and role of SDCBP2.

**Results:**

The multivariate Cox regression confirmed SDCBP2 as an independent prognostic factor. The ROC curve based on SDCBP2 expression showed a strong predictive power for the prognosis of LUAD patients at 1, 3, and 5 years. The GSEA and GO/KEGG analysis linked SDCBP2 to ferroptosis and cell cycle pathways. Next, the *in vitro* results revealed that knockdown of SDCBP2 induced G0/G1 phase arrest and apoptosis, and inhibited the proliferation and migration of LUAD cells. Meanwhile, knockdown of SDCBP2 reduced glutathione (GSH) levels and enhanced the cellular level of ROS. Furthermore, we found a correlation between the expression of SDCBP2 and SLC7A11, and patients with concurrent high expression of both exhibited a poorer prognosis, although the regulatory relationship between the two genes remains to be further investigated.

**Discussion:**

This study demonstrates that SDCBP2 promotes tumor progression and is a novel ferroptosis-related prognostic biomarker for LUAD.

## 1 Introduction

Lung cancer is currently the most commonly diagnosed cancer type worldwide and is the leading cause of cancer-related deaths globally. With changes in people’s lifestyles, lung adenocarcinoma (LUAD) has replaced squamous cell carcinoma as the most common pathological type, accounting for approximately 40% of all lung cancers (1). Unlike other types of lung cancer, lung adenocarcinoma exhibits high genetic heterogeneity, and personalized targeted therapies have provided substantial benefits for patients with specific molecular subtypes of lung adenocarcinoma (2, 3). However, the antitumor efficiency in lung adenocarcinoma patients is occasionally limited by target resistance and apoptosis escape, leading to tumor recurrence and poor prognosis. The exploration for specific molecular targets and the development of non-apoptotic induced drugs remain a high priority in the treatment of lung adenocarcinoma (4–6).

Ferroptosis is a novel type of regulated cell death (RCD), characterized by the accumulation of iron-dependent lipid peroxides leading to membrane rupture and cell death (7). In recent years, ferroptosis has garnered significant attention as an alternative target to apoptosis in cancer therapy. It has been found that ferroptosis is closely associated with every stage of LUAD, including initiation, proliferation, and progression (8, 9). However, due to the long-term exposure of lung tissue to high oxygen concentrations, the induction threshold for ferroptosis in lung cancer cells increases, serving the purpose of protecting themselves from oxidative stress damage (10). Therefore, identifying potential molecular targets that can promote ferroptosis is key to achieving ferroptosis-based lung cancer treatment (11, 12).

In this study, we constructed a risk model using ferroptosis regulator genes reported in previous literature to screen genes related to lung adenocarcinoma prognosis. We discovered that the gene SDCBP2, as a novel lung cancer biomarker, is closely related to ferroptosis. Through bioinformatics analysis and *in vitro* experiments, we demonstrated that SDCBP2, as a potential therapeutic target, is an independent prognostic biomarker for LUAD, and inhibiting its expression may suppress tumor progression by affecting the cell cycle, apoptosis and ferroptosis.

## 2 Materials and methods

### 2.1 Data acquisition

We downloaded pan-cancer transcriptome analysis data, clinical information, and prognostic data (including 20,000 primary cancer samples and corresponding non-carcinoma samples from 33 types of cancers) from the TCGA database (https://portal.gdc.cancer.gov/). We also downloaded the GSE72094 dataset (containing tumor samples from 442 lung adenocarcinoma patients) from the GEO database (https://www.ncbi.nlm.nih.gov/geo/). We used the R-sva package to standardize the data from the TCGA-LUAD and the GSE72094 cohorts to remove batch effects.

### 2.2 Risk model construction for ferroptosis in lung adenocarcinoma

A total of 65 ferroptosis regulator genes were identified from previously published literature (13, 14). In the TCGA-LUAD cohort, the expression profiles of ferroptosis regulator genes between tumor and normal tissues were compared. Differentially expressed genes (DEGs) were selected using the R-limma package with the screening criteria of |log2Fold Change| > 0.585 and FDR < 0.05. Subsequently, the R-survival package was used to perform Cox regression analysis on DEGs to select prognosis-related DEGs and to construct a prognostic risk model using Lasso (least absolute shrinkage and selection operator) regression analysis, with the optimal penalty parameter λ determined by 10-fold cross-validation based on minimum deviance. The risk model score was used to divide the samples in TCGA-LUAD into high-risk and low-risk groups, and to compare gene expression differences between groups to select differentially expressed genes in TCGA cohort (TCGA-DEGs). Additionally, the same risk score was applied to the GSE72094 cohort for external validation and to select differentially expressed genes in GES72094 cohort (GEO-DEGs).

### 2.3 The expression and prognostic value of SDCBP2 in pan-cancer and lung adenocarcinoma

The TIMER database (https://cistrome.shinyapps.io/timer/) was used to analyze the expression profile of SDCBP2 in pan-cancer. Cox regression analysis was employed to assess the impact of SDCBP2 expression on prognosis in different types of cancer. LUAD patients were divided into high and low expression groups based on the median expression value of SDCBP2 (3.619 for TPM). The expression of SDCBP2 and its correlation with clinical and pathological characteristics of LUAD patients were analyzed in the TCGA-LUAD cohort. To determine the independent prognostic value of SDCBP2 in LUAD, univariate and multivariate Cox analyses were conducted, and the proportional hazards assumption was assessed using the global test of Schoenfeld residuals. Furthermore, the expression status of SDCBP2 in single-cell databases was analyzed on the TISCH2 online database (http://tisch.comp-genomics.org/).

### 2.4 Genome Ontology/Kyoto Encyclopedia of Genes and Genomes enrichment analyses of the DEGs and gene set enrichment analysis

The expression profiles of the high and low SDCBP2 expression groups were compared to identify the DEGs via the R-limma package (15), according to the criteria of |log2Fold Change| > 1.0 and FDR < 0.05. The GO term and KEGG pathway enrichment analyses of the DEGs were carried out by using R software. The GO analysis included biological process (BP), cellular component (CC) and molecular function (MF) terms categories. Additionally, we conducted GSEA (16) in the TCGA-LUAD cohort. The annotated reference gene set is “c2.cp.all.v2022.1.Hs.symbols.gmt”.

### 2.5 Associations of the expression levels of SDCBP2 and ferroptosis regulator genes in LUAD

We analyzed the differences in expression levels of ferroptosis regulator genes between high and low SDCBP2 expression groups in the TCGA-LUAD and the GSE72094 cohorts, as well as their correlation with SDCBP2 expression. Furthermore, we screened for ferroptosis regulator genes that are closely related to SDCBP2.

### 2.6 *In vitro* experiments

#### 2.6.1 Cell culture and siRNA transfection

Two LUAD cell lines (A549 and PC-9), 10% fetal bovine serum, and 0.25% trypsin were purchased from Procell (Wuhan, China). Cells were cultured in RPMI 1640 medium (Servicebio, China) supplemented with 10% fetal bovine serum. All cell lines were maintained in a sterile, contamination-free incubator at 37 °C and 5% CO2.

SDCBP2-siRNAs were constructed and synthesized by GenePharma (Shanghai, China) for SDCBP2 knockdown experiments. After the cultured cell lines entered the logarithmic growth phase, SiRNA was transiently transfected into LUAD cell lines using jetPRIME transfection reagent (Polyplus, France). The siRNA sequences are shown in **Supplementary Table S1**. The transfection efficiency of SDCBP2 was detected using qPCR and western blotting, and subsequent experiments were conducted.

#### 2.6.2 RNA extraction and real-time quantitative PCR assays

Total RNA was extracted from LUAD cell using the Trizol method, and the concentration of RNA was measured using an ultraviolet spectrophotometer. The extracted total RNA was reverse transcribed into cDNA using PrimeScript RT Master Mix (RR036A, Takara). Real-time PCR was performed on a Bio-Rad CFX Connect Real-Time System using TB Green Premix Ex Taq (RR820A, Takara). β-actin was used as the internal reference for mRNA qPCR. The 2^^(-ΔΔCT)^ method was used to analyze the relative expression levels of genes. The sequences of primers used were shown in **Supplementary Table S2**.

#### 2.6.3 Western blotting

Total proteins were extracted with RIPA buffer (#R0020, Solarbio, China). The extracted proteins were resolved by SDS polyacrylamide gel electrophoresis and transferred to a nitrocellulose membrane. After blocking in 5% nonfat milk for 2 h, the membranes were incubated overnight at 4 °C with primary antibodies recognizing SDCBP2 (1:1000 dilution; Cat No. 10407-1-AP, Proteintech, USA), SLC7A11 (1:2000 dilution; Cat No. 10407-1-AP, Proteintech, USA) and β-actin (1:20000 dilution; Cat No. 81115-1-RR, Proteintech, USA). After incubation with secondary antibodies (1:5000 dilution; CW0103, Cowin Biotech, China), the protein bands were visualized by chemiluminescence using a Bio-Rad ChemiDoc MP Imaging System (Bio-Rad, USA).

#### 2.6.4 Cell Counting Kit-8 assay

LUAD cells (1×10^4^/mL) were seeded into a 96-well plate, followed by transfection of si-SDCBP2 and si-NC into the experimental and control groups, respectively. Cell counting kit-8 (Coolaber, China) was mixed with 10% complete medium to form a 10% CCK-8 mixture, and 100 μL of the mixture per well was added to A549 and PC-9 cells. Before each absorbance measurement, the cells were incubated in a sterile incubator for 2 hours, and the optical density (OD) was measured at 0 hours, 24 hours, 48 hours, 72 hours, and 96 hours. The highest and lowest values were discarded, and the remaining values were averaged to obtain the proliferation level.

#### 2.6.5 Colony formation assay

After resuspension, LUAD cells were seeded into a 6-well plate at a density of 1000 cells per well and cultured for one week. Then, the cells were fixed with 4% paraformaldehyde for 30 minutes and stained with 0.5% crystal violet for 30 minutes. After washing three times and air-drying, the plates were imaged by camera, and colonies with more than 50 cells were manually counted.

#### 2.6.6 Wound healing assay

LUAD cells were cultured in 6-well plates and grown to 100% confluency. Artificial homogeneous wounds were created with a 10 μl pipette tip. After washing with PBS to remove detached debris, serum-free medium was added to the six-well plates. The wounded areas were photographed under a microscope at 0 hours and after incubation for 24 hours in a cell culture incubator. The data were quantified using the following formula: Migration rate % = (Initial scratch area - Scratch area at time t)/Initial scratch area × 100%.

#### 2.6.7 Transwell migration assay

LUAD cells were resuspended in serum-free medium and seeded into the upper chamber of a 24-well transwell (8 μm, Corning, USA) at a density of 20,000 cells per well. The lower chamber was filled with medium supplemented with 10% FBS. After 24 hours, cells were fixed with 4% paraformaldehyde for 30 minutes, followed by staining with 5% crystal violet for 10 minutes. Unmigrated cells were removed by wiping with a cotton swab, and then stained cells were imaged using a fluorescence microscope system (Olympus, Japan).

#### 2.6.8 Cell cycle assay

LUAD cells were harvested at 70-80% confluency using 0.05% trypsin and washed with pre-chilled PBS. The cell pellet was resuspended in pre-chilled 70% ethanol and fixed overnight at -20 °C. The following day, the cells were washed again with pre-chilled PBS and resuspended in PI/RNase Staining Buffer (Cat. No. 550825, BD Biosciences, USA). Before analysis, the mixture was incubated in the dark at 37 °C for 30 minutes. Cell cycle distribution was detected using a NovoCyte Flow Cytometer (Agilent, USA). Different cell cycle stages were analyzed using NovoExpress software (version 1.6.2).

#### 2.6.9 Cell apoptosis assay

LUAD cell apoptosis was detected using the APC-Annexin V/PI Cell Apoptosis Kit (A6030L, UElandy, China). Cells were harvested at 70-80% confluency using EDTA-free trypsin and washed with pre-chilled PBS. The cell pellet was resuspended in binding buffer, and then 10^5^ cells were collected. Subsequently, 5 μl of Annexin V-APC and 5 μl of PI were added and mixed gently. The samples were incubated in the dark at room temperature for 15 minutes. The apoptosis rate was detected using a NovoCyte Flow Cytometer (Agilent, United States), and the obtained data were analyzed using NovoExpress software (version 1.6.2).

#### 2.6.10 Measurement of reactive oxygen species and GSH levels

According to the manufacturer’s instructions, 2,7-dichlorofuorescindiacetate (DCFH-DA) reactive oxygen species fluorometric assay kit (E-BC-K138-F, Elabscience, China) was used to detect the intracellular ROS production of LUAD cells. The accumulation of DCF was observed using a fluorescence microscope, and quantitatively assessed using ImageJ software. The levels of glutathione (GSH) were analyzed using a GSH colorimetric assay kit (Elabscience, E-BC-K030-M,China). The GSH levels were measured against the standard calibration curves based on absorbances at 405 nm with the microplate reader.

#### 2.6.11 Immunohistochemical staining and scoring

We used tissue samples from LUAD patients to assess the expression level of SDCBP2 and SLC7A11 through IHC staining. Samples from a total of 15 LUAD patients who underwent lung cancer operation at the First Affiliated Hospital of Zhengzhou University between May 2023 and February 2024, were used to construct tissue microarrays (TMA). The TMA were incubated with SDCBP2 monoclonal antibody (1:500 dilution) and SLC7A11 (1:200 dilution) at 4 °C overnight. The TMAs were washed the next morning and incubated with a secondary antibody (GB23303, Servicebio, China) for 30 min at 37 °C. Diaminobenzene was used as the chromogen, and hematoxylin was used as the nuclear counterstain. The immunostaining intensity was scored based on the H‐score [=(percentage of weak intensity×1)+(percentage of moderate intensity×2)+(percentage of strong intensity × 3)].

### 2.7 Statistical analysis

Raw mRNA expression data downloaded from the online database were normalized by [log2 (data+1)] for further statistical analysis. Statistical methods including ANOVA, Student’s t-test, Chi-square test, log-rank test, Pearson’s and Spearman’s correlation analysis were used to analyze the relationship between variables. Survival analysis was performed using the Kaplan-Meier method, and comparisons were made using the log-rank test. Univariate and multivariate analyses were performed using the Cox regression model. All experiments were repeated three times and data are expressed as the mean ± standard deviation. Two-group comparisons were analyzed using the unpaired Student’s t-test, whereas one-way ANOVA followed by Tukey’s *post-hoc* test was use for multiple comparisons. Data processing and statistical analysis were carried out using R (v4.2.2) and Strawberry Perl (v.5.32.1.1).Based on the changes observed in the image, ImageJ is used for graphics calculations. Unless otherwise specified, P-values < 0.05 were considered to indicate statistical significance, and all the P-values were calculated as two-tailed tests.

## 3 Results

### 3.1 Construction of ferroptosis risk model and screening of hub genes in LUAD

**Figure 1:**
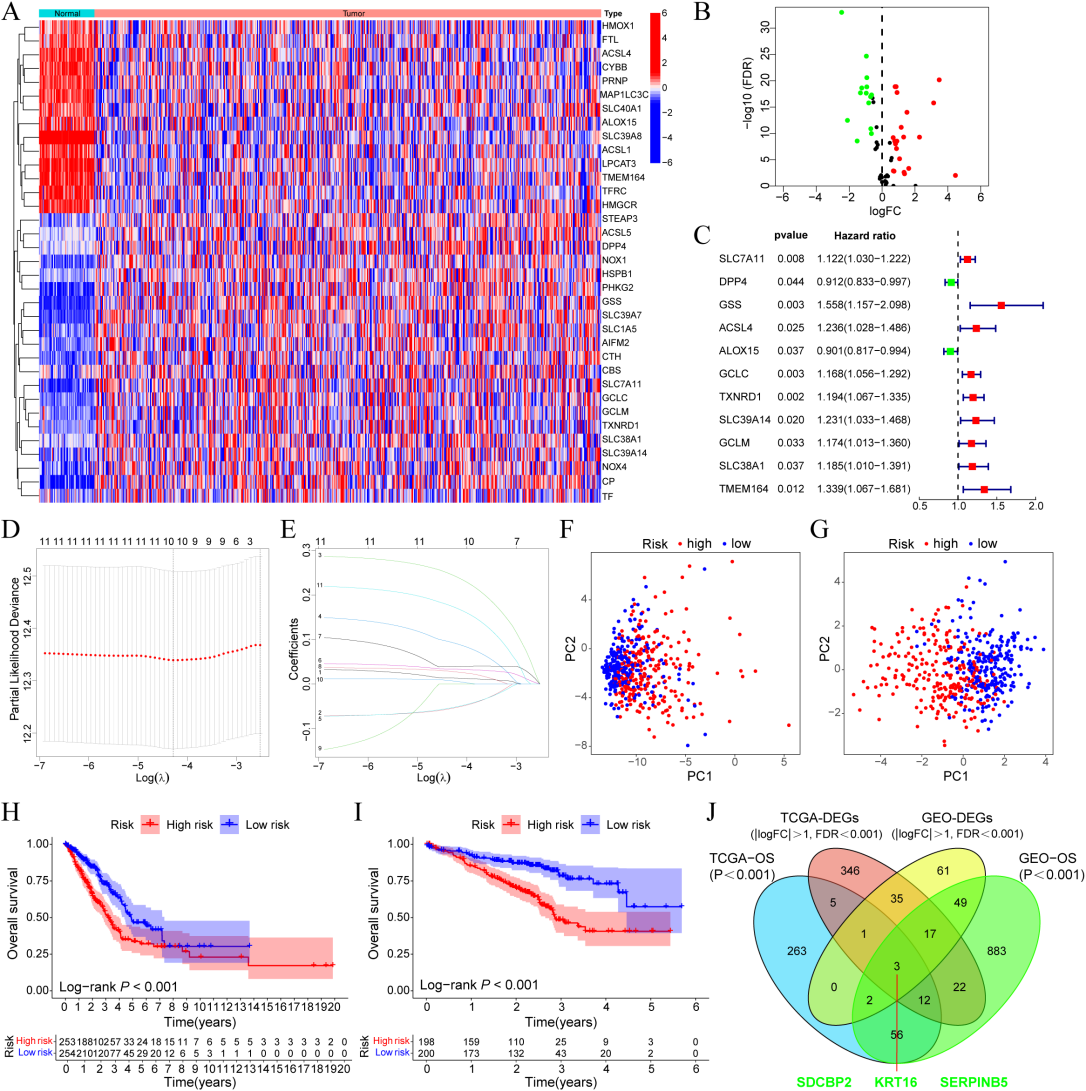
SDCBP2 is a novel ferroptosis-related biomarker in LUAD. **(A)** Expression heatmap of DEGs between tumor and normal samples. **(B)** Volcano plot of DEGs. **(C)** Forest plot of prognostic-related DEGs. **(D, E)** Lasso regression analysis to identify 10 risk genes. **(F, G)** PCA analysis plots before **(F)** and after **(G)** modeling. **(H, I)** OS differences between high and low-risk groups in the TCGA **(H)** and GEO **(I)** databases, respectively. **(J)** Intersection analysis of DEGs and prognostic-related genes between GEO and TCGA cohorts.

In the TCGA-LUAD cohort, the expression profiles of ferroptosis regulator genes between tumor and normal tissues were compared, and 35 DEGs were selected based on the criteria of |log2Fold Change| > 0.585 and FDR < 0.05, including 21 upregulated genes and 14 downregulated genes (**Figures 1A, B**). Univariate Cox regression analysis was performed on these DEGs, and 11 prognostic-related DEGs were selected (**Figure 1C**). We constructed a prognostic risk model based on these 11 genes through Lasso regression analysis (**Figures 1D, E**), and ultimately, 10 prognostic-related DEGs with the optimal λ value were included (The risk score = GSS×0.244 + TMEM164×0.174 + ACSL4×0.093 + TXNRD1×0.039 + GCLC×0.034 + SLC39A14×0.028 + SLC7A11×0.014 + SLC38A1×0.007 - DPP4×0.054 - ALOX15×0.053). Patients were divided into high-risk and low-risk groups based on the median risk score. Principal component analysis (PCA) showed that, compared to all ferroptosis regulator genes (**Figure 1F**), this model could effectively differentiate patients with high and low prognostic risk (**Figure 1G**). Survival analysis in the TCGA-LUAD cohort indicated that patients in the high-risk group had poorer OS (P < 0.001, **Figure 1H**), which was also verified in GSE72094 (**Figure 1I**). Furthermore, we compared the gene expression differences between high and low-risk groups in the two cohorts, identified the corresponding DEGs (TCGA-DEGs and GEO-DEGs), and intersected these with the prognostic-related genes from each cohort to obtain three hub genes—SDCBP2, DKK1, and SERPINB5 (**Figure 1J**). Among them, SDCBP2, as a novel tumor biomarker, has not yet been reported in the literature.

### 3.2 Pan-cancer analysis of SDCBP2 expression level

**Figure 2:**
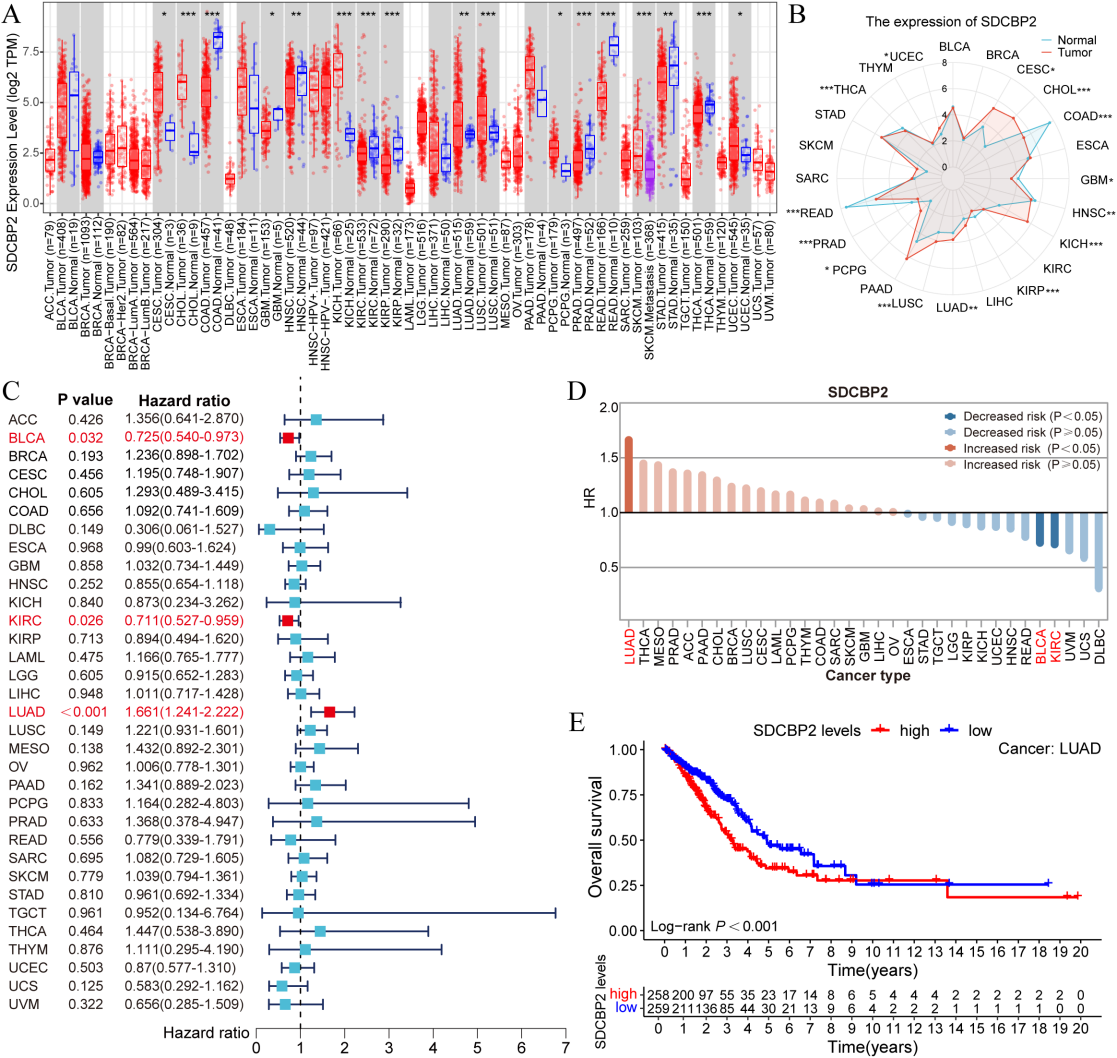
Pan-cancer analysis of SDCBP2 expression. **(A, B)** Pan-cancer expression data for SDCBP2 in TIMER database. **(C)** Forest plot for survival analysis of patients stratified by SDCBP2 expression. **(D)** Risk degree of high SDCBP2 expression in various cancers. **(E)** Correlation between SDCBP2 expression and OS in LUAD patients. *P < 0.05; **P < 0.01; ***P < 0.001.

We utilized the TIMER database to analyze the expression of SDCBP2 in the TCGA pan-cancer dataset. We found that the expression of SDCBP2 was significantly upregulated in Cervical Cancer (CESC), Bile Duct Cancer (CHOL), Kidney Chromophobe (KICH), Lung Adenocarcinoma (LUAD), Lung Squamous Cell Carcinoma (LUSC), Pheochromocytoma & Paraganglioma (PCPG), and Endometrioid Cancer (UCEC), and significantly downregulated in Colon Cancer (COAD), Glioblastoma (GBM), Head and Neck Cancer (HNSC), Kidney Papillary Cell Carcinoma (KIRP), Prostate Cancer (PRAD), Rectal Cancer (READ), and Thyroid Cancer (THCA) (**Figures 2A, B**). We then assessed the impact of SDCBP2 on overall survival (OS) in various cancers (**Figures 2C, D**) and found that SDCBP2 is a prognostic risk gene only in LUAD (HR = 1.661, 95%CI: 1.241-2.222, P < 0.001), while it is a prognostic protective gene in BLCA and KIRC (HR = 0.725, 95%CI: 0.540-0.973, P = 0.032; HR = 0.711, 95%CI: 0.527-0.959, P = 0.026). Survival curves also indicated that patients with high SDCBP2 expression in the TCGA-LUAD cohort have poorer OS (P < 0.001, **Figure 2E**).

### 3.3 Expression levels, clinical significance, and prognostic value of SDCBP2 in LUAD

**Figure 3:**
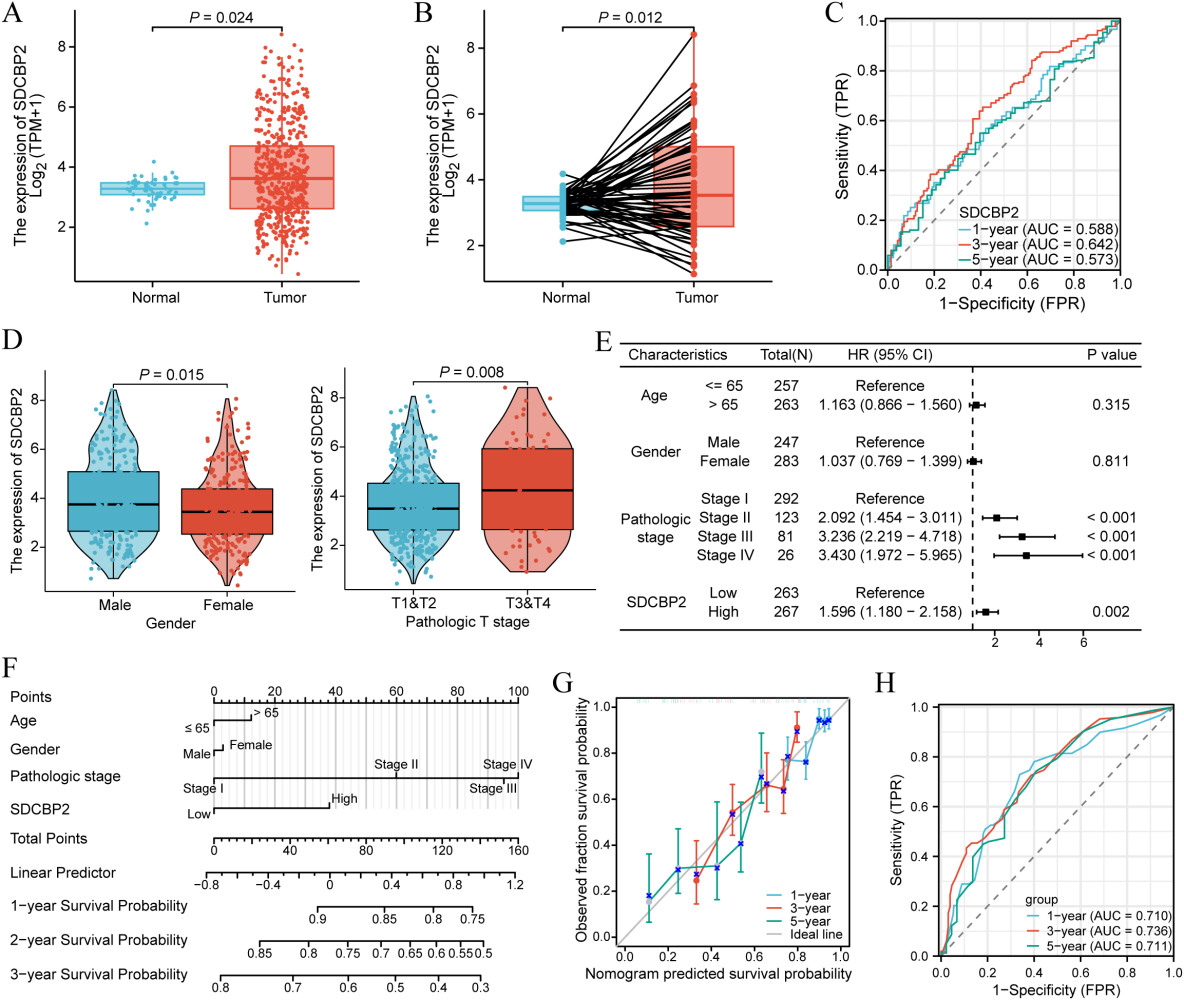
Expression and prognostic value of SDCBP2 in LUAD. **(A)** Expression of SDCBP2 in the TCGA-LUAD cohort. **(B)** Expression of SDCBP2 in TCGA-LUAD tissue and paired normal tissue. **(C)** Prognostic ROC curve analysis based on SDCBP2 expression levels. **(D)** Correlation between SDCBP2 expression and gender, pathological staging. **(E)** Multivariable Cox regression analysis based on SDCBP2 expression and major clinicopathological characteristics. **(F)** Nomogram analysis of clinicopathological parameters and SDCBP2 expression. **(G)** Calibration curves of 1, 3, 5 years of SDCBP2. **(H)** Prognostic ROC curve analysis based on the nomogram risk score.

In the TCGA-LUAD cohort, we found that the expression level of SDCBP2 in tumor samples was significantly higher than in normal tissue in both unpaired samples (P = 0.024, **Figure 3A**) and paired samples (P = 0.012, **Figure 3B**) (P < 0.05). The receiver operating characteristic (ROC) curve showed that the expression of SDCBP2 has certain predictive performance for the prognosis diagnosis of LUAD patients (**Figure 3C**). Subsequently, we analyzed the correlation between SDCBP2 expression and clinicopathological characteristics (**Figure 3D**, **Supplementary Figure S1**). The results showed that the upregulation of SDCBP2 expression is closely related to gender (P = 0.015) and Tumor stage (P = 0.008). To determine whether SDCBP2 expression can serve as an independent prognostic factor for LUAD, we included the main clinical pathological characteristics (including age, gender, pathological staging) along with SDCBP2 expression in a multivariable Cox regression and found that SDCBP2 expression level (HR = 1.596, 95%CI: 1.180-2.158, P = 0.002) and pathological staging (P < 0.001) are both independent prognostic factors for LUAD (**Figure 3E**). Subsequently, we integrated the above factors to construct a prognostic nomogram to predict the prognosis of LUAD patients at 1, 3, and 5 years (**Figure 3F**), and the calibration curves showed good consistency between the nomogram predictions and the actual observed results (**Figure 3G**). The ROC curve showed that the nomogram has strong predictive power for the prognosis of LUAD patients at 1, 3, and 5 years(AUC = 0.710, 0.736, and 0.711, **Figure 3H**).

**Figure 4:**
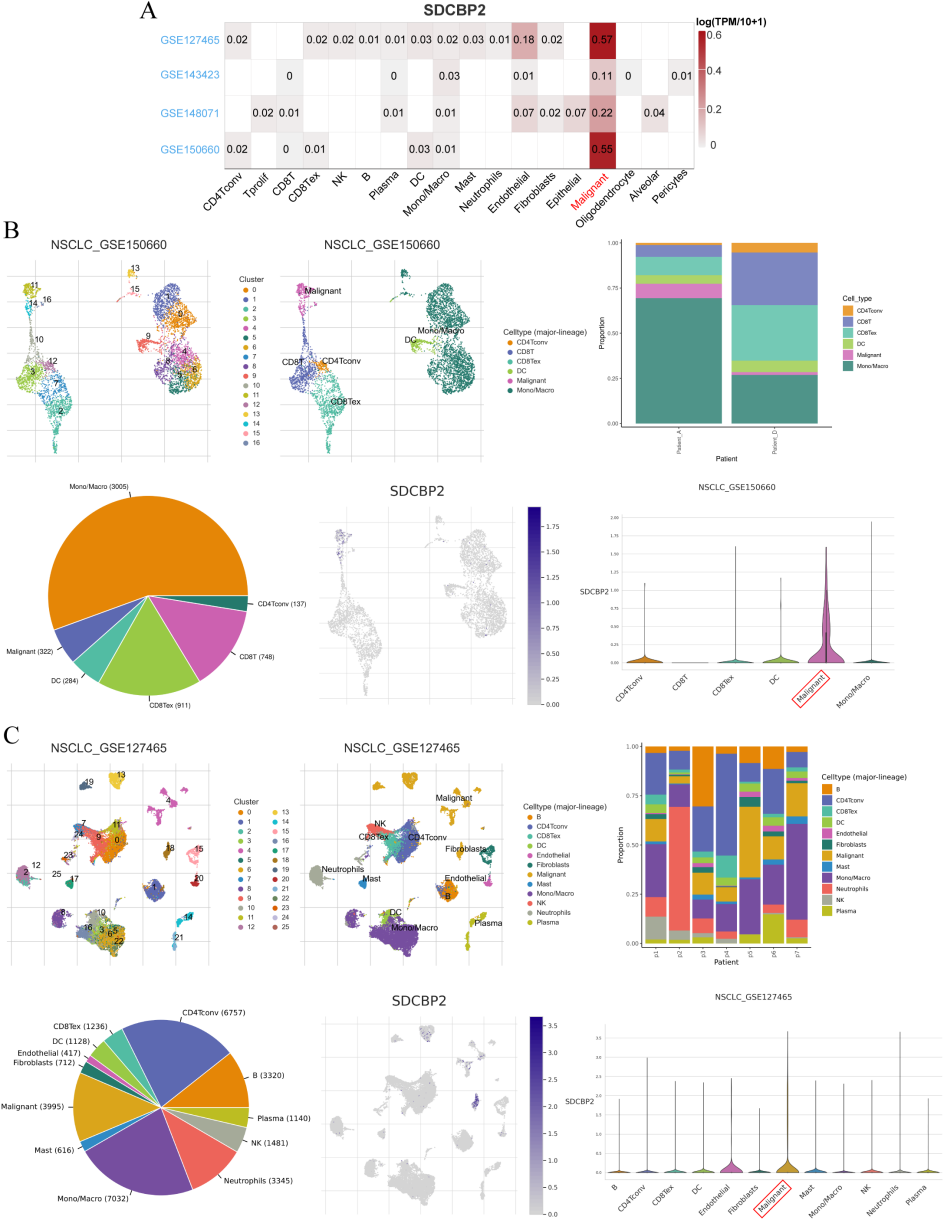
Expression status of SDCBP2 in TISCH2-NSCLC single-cell database. **(A)** Heatmap of SDCBP2 expression distribution in different cell lines. **(B)** The expression of SDCBP2 is enriched in malignant cell type in GSE150660 database; **(C)** SDCBP2 expression is enriched in malignant cell type in GSE127465 database.

Additionally, to further explore the expression of SDCBP2 in different cell types among LUAD patients, we investigated the expression status of SDCBP2 in the NSCLC-related single-cell databases on the TISCH2 website. In all NSCLC-related single-cell databases, SDCBP2 expression was significantly enriched in malignant cells (**Figure 4A**), and we conducted further analysis on the datasets with the highest enrichment, GSE150660 and GSE127465 (**Figures 4B, C**), finding that the expression of SDCBP2 showed a clear distributional selectivity.

### 3.4 GO/KEGG enrichment analysis of the DEGs and GSEA

**Figure 5:**
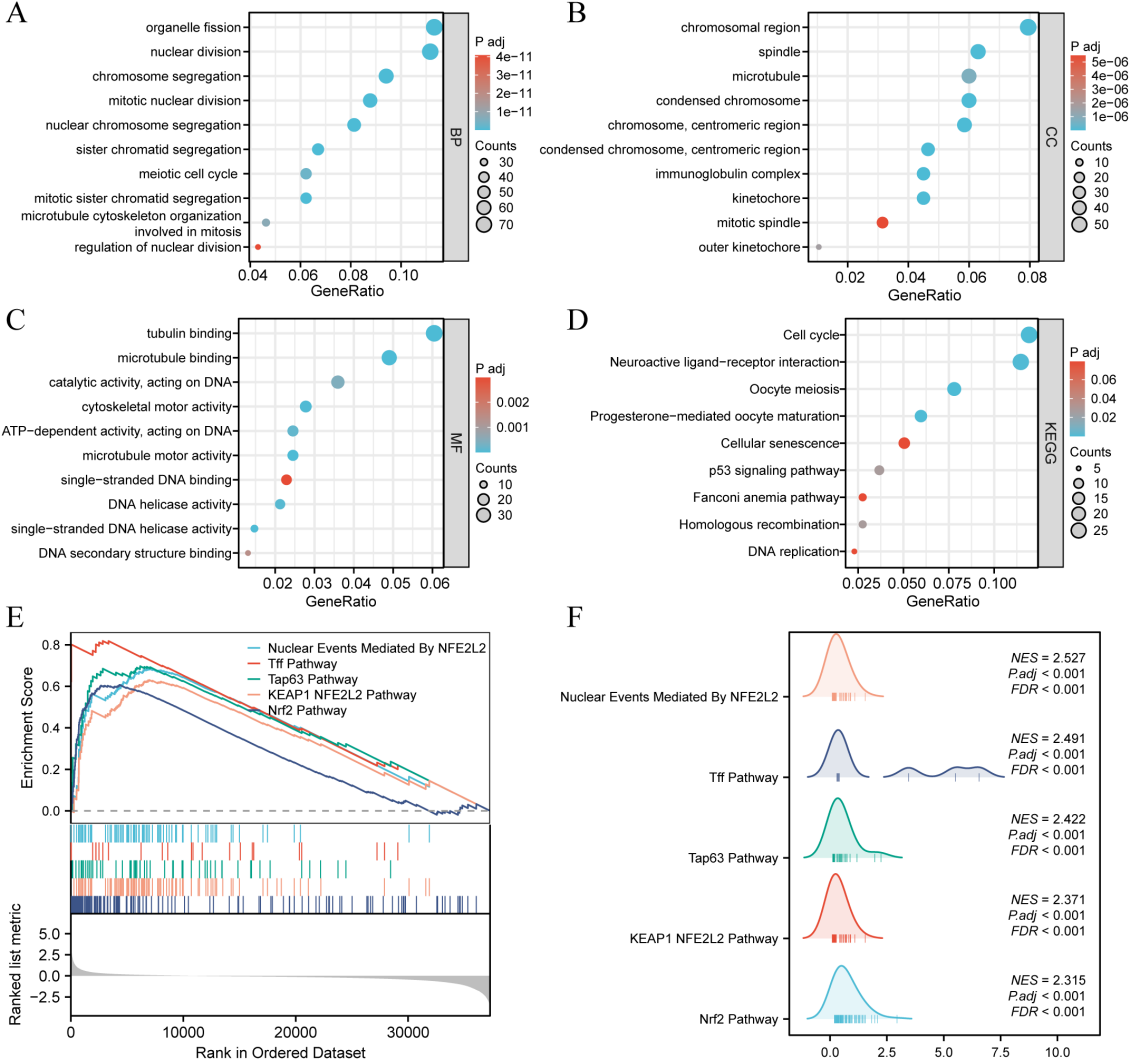
Enrichment analysis. **(A-D)** GO and KEGG enrichment analysis of DEGs between high and low SDCBP2 expression groups. **(E, F)** Enrichment of genes in annotated gene sets by GSEA.

According to the criteria of |log2 Fc| > 1.0 and FDR < 0.05, a total of 871 differentially expressed genes (DEGs) were identified between the high and low SDCBP2 expression groups, including 432 upregulated genes and 439 downregulated genes. GO term annotation indicated that these genes are primarily involved in biological processes such as cell division, chromosome behavior, microtubule activity, and DNA metabolism (**Figures 5A–C**). KEGG pathway analysis revealed that these genes are mainly involved in the cell cycle, p53 signaling pathway, DNA replication, and cellular senescence (**Figure 5D**). To further explore the signaling pathways associated with SDCBP2 expression, we performed GSEA, and **Figures 5E, F** shows the most significant 5 pathways related to molecular signaling pathway in the high expression group. The results showed that patients with high expression of SDCBP2 may exhibit activation of pathways such as the “KEAP1-NFE2L2 pathway”, “Tff pathway” and “Tap63 pathway”.

### 3.5 Downregulation of SDCBP2 inhibits the proliferation and migration of LUAD cells

**Figure 6:**
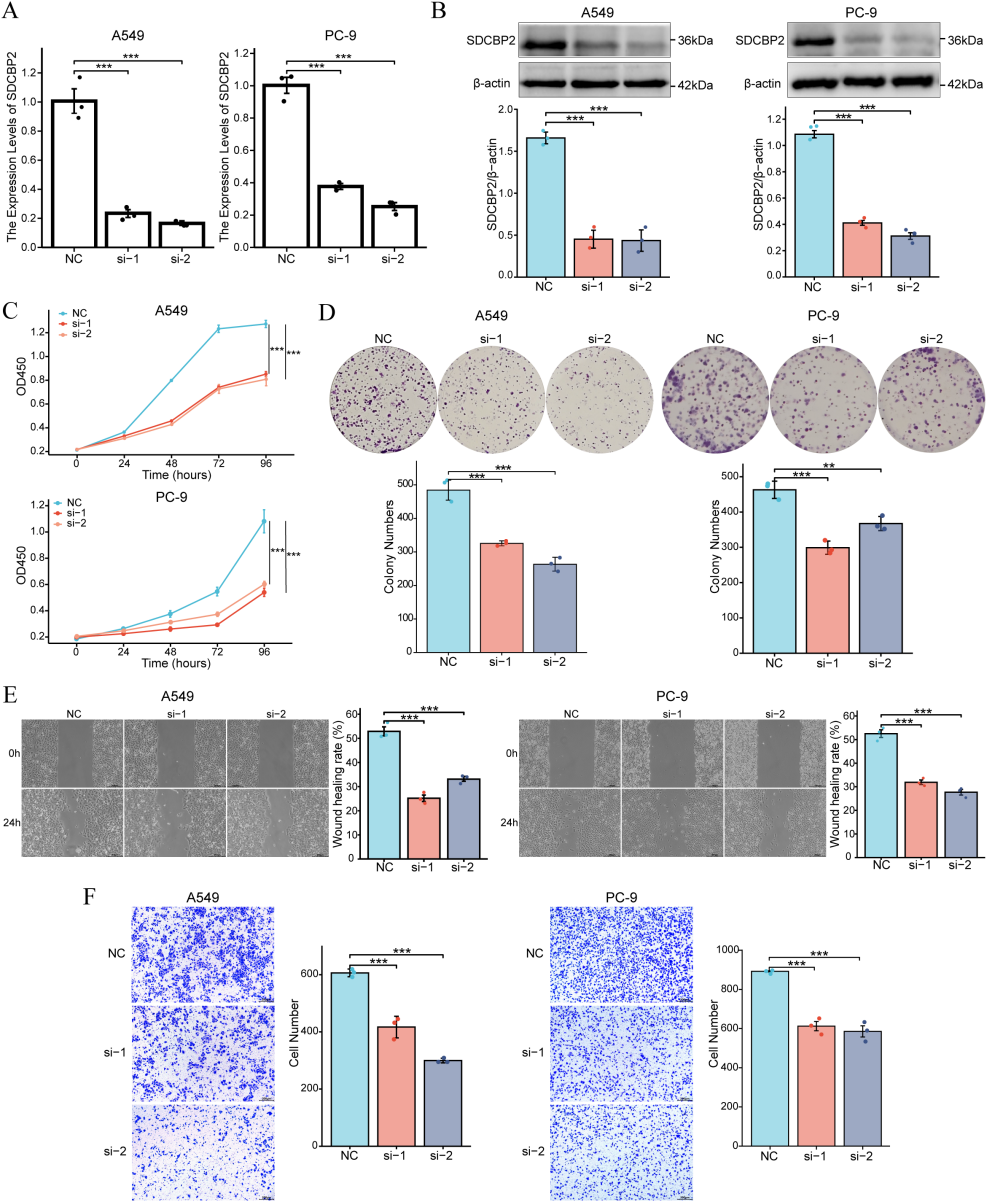
Downregulation of SDCBP2 inhibits the proliferation and migration of LUAD cell lines (A549 and PC-9). **(A, B)** The effect of SDCBP2 siRNAs in LUAD cell lines was assessed by qRT-PCR **(A)** and Western Blot **(B)**. **(C, D)** CCK-8 assay **(C)** and colony formation assay **(D)** results show that knocking down SDCBP2 levels can inhibit the proliferation of LUAD cells. **(E, F)** Wound healing assay and Transwell assay show that downregulation of SDCBP2 can inhibit the migration of LUAD cells. *P < 0.05; **P < 0.01; ***P < 0.001.

To further investigate the role of SDCBP2 in LUAD, *in vitro* experiments were conducted. We knocked down the expression of SDCBP2 in LUAD cell lines (A549 and PC-9) by transfecting SDCBP2 siRNA (si-1 and si-2). Compared with negative control (NC) group, the expression of si-1 group and si-2 group were significantly suppressed (**Figures 6A, B**). CCK-8 assays indicated that SDCBP2 knockdown significantly inhibited cell growth (**Figure 6C**). Similarly, colony formation assays showed that the clonogenic ability of LUAD cells decreased significantly after SDCBP2 knockdown (**Figure 6D**). Wound healing assays and Transwell assays demonstrated that SDCBP2 knockdown significantly reduced the migration ability of LUAD cells (**Figures 6E, F**). In summary, the knockdown of SDCBP2 inhibits the proliferation and migration of LUAD cells.

### 3.6 Downregulation of SDCBP2 induces G1 phase cell cycle arrest and apoptosis in LUAD cells

**Figure 7:**
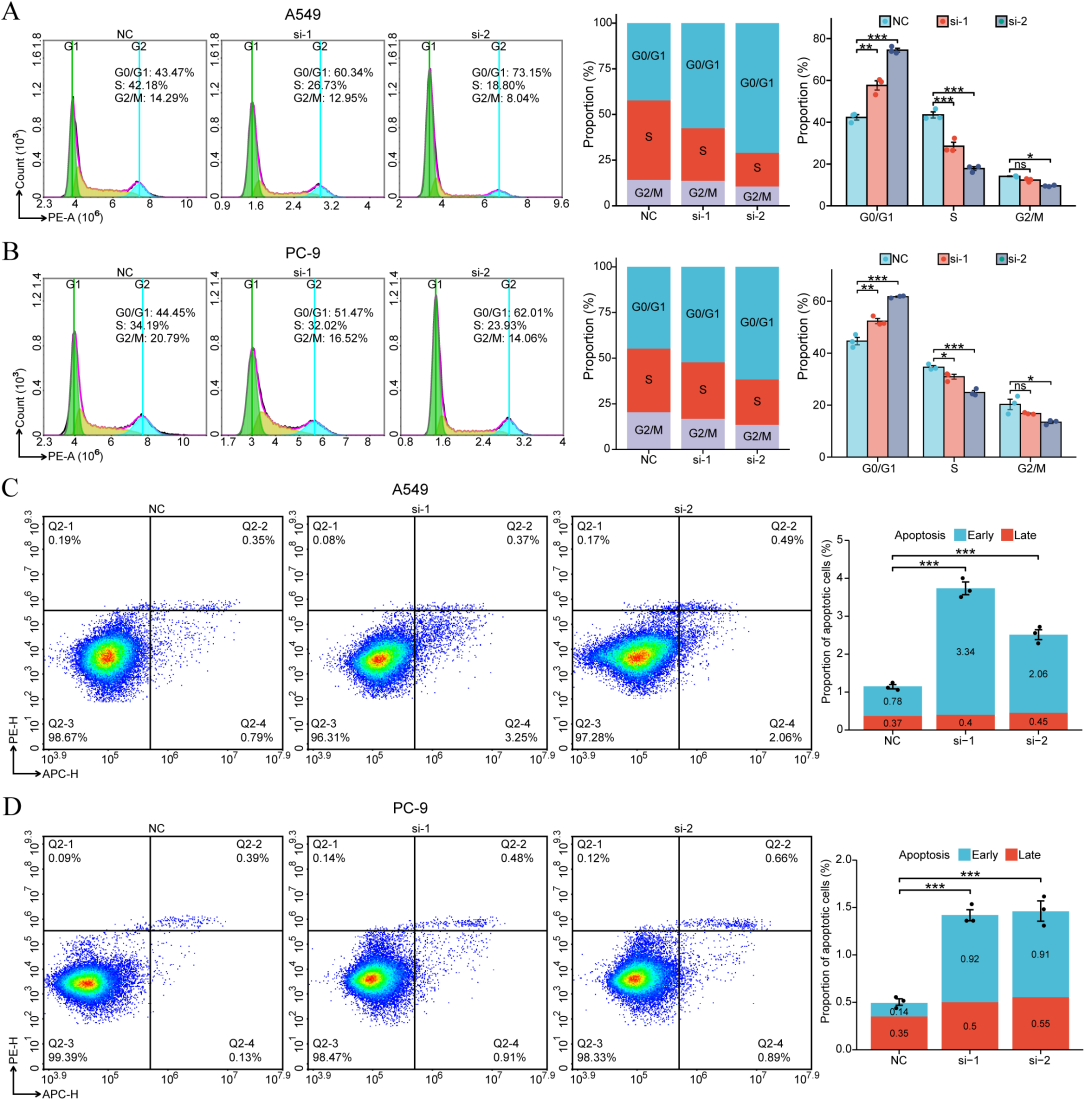
SDCBP2 regulates cell cycle and apoptosis in LUAD cell lines (A549 and PC-9). **(A, B)** Flow cytometry was used to determine the proportions of cells in G0/G1, S, and G2/M phases following transfection with SDCBP2 siRNA in A549 cells **(A)** and PC-9 cells **(B)**. **(C, D)** The impact of SDCBP2 knockdown on apoptosis in A549 cells **(C)** and PC-9 cells **(D)** was analyzed by flow cytometry apoptosis assay. *P < 0.05; **P < 0.01; ***P < 0.001.

As stated earlier, GO/KEGG enrichment analysis results suggest that the expression of SDCBP2 may be closely related to the regulation of the cell cycle and cellular senescence. Flow cytometry analysis of cell cycle changes revealed that after SDCBP2 knockdown, the proportion of A549 and PC-9 cells in the G0/G1 phase significantly increased, while the proportion of cells in the S phase correspondingly decreased, indicating G1 phase arrest (**Figures 7A, B**). Furthermore, we also used flow cytometry to detect changes in the incidence of apoptosis after SDCBP2 knockdown and found that downregulation of SDCBP2 increased apoptosis in A549 and PC-9 cells (**Figures 7C, D**). The results summarized above indicate that downregulation of SDCBP2 can induce apoptosis and G1 phase cell cycle arrest in LUAD cells.

### 3.7 Associations of SDCBP2 expression with ferroptosis in LUAD

**Figure 8:**
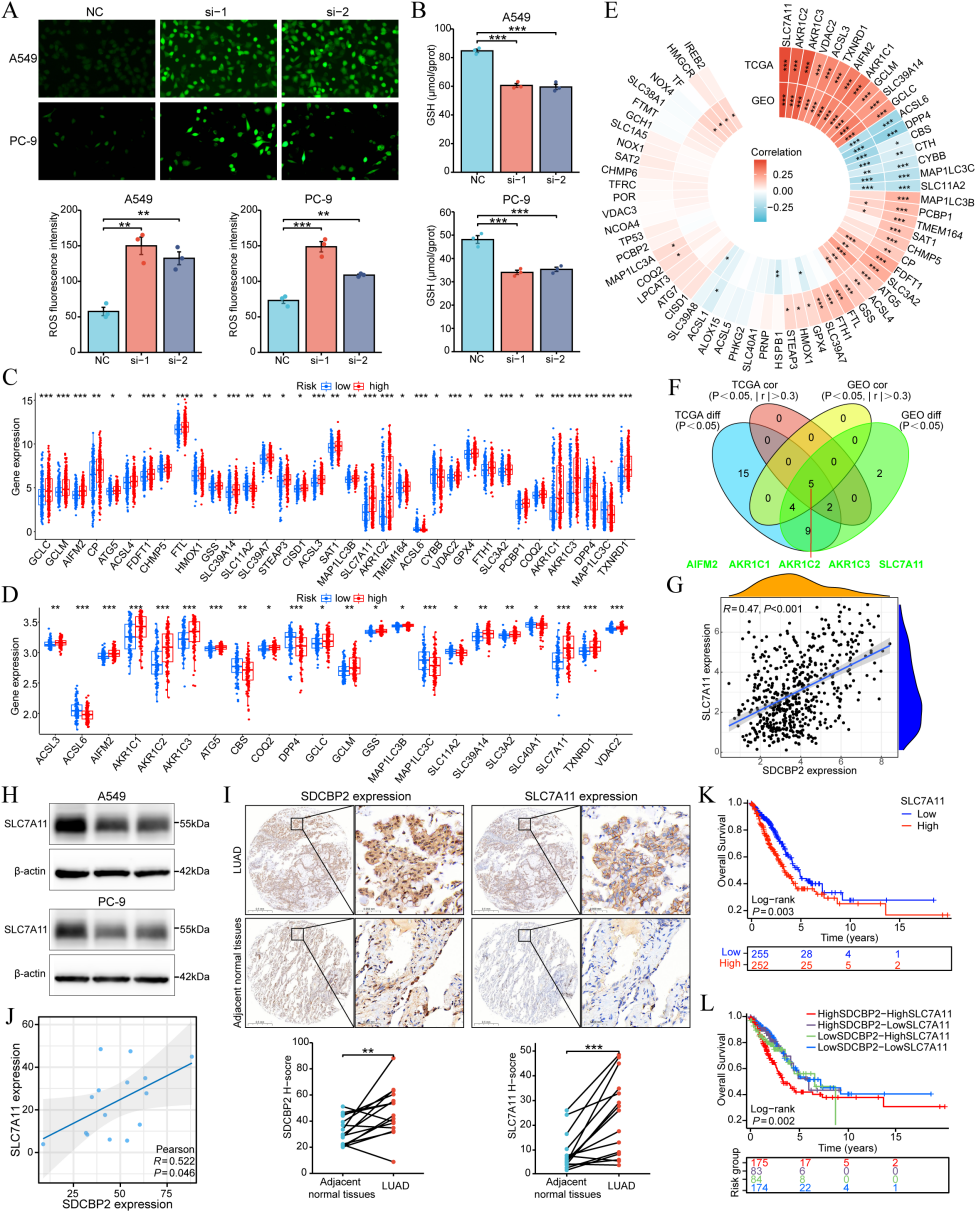
Association between SDCBP2 expression levels and ferroptosis in LUAD. **(A, B)** Intracellular ROS **(A)** and GSH **(B)** levels in A549 and PC-9 cell lines. **(C, D)** Expression difference analysis of ferroptosis regulatory genes grouped by SDCBP2 expression in TCGA-LUAD **(C)** and GSE72094 cohorts **(D)**. **(E)** Correlation analysis between SDCBP2 expression and ferroptosis regulatory genes in TCGA-LUAD and GSE72094 cohorts. **(F)** Intersection of differential and correlation analyses. **(G)** Correlation analysis between SDCBP2 and SLC7A11 expression in TCGA-LUAD cohort. **(H)** The protein level of SLC7A11 in in A549 and PC-9 cell lines. **(I, J)** IHC scoring **(I)** and H-score correlation analysis **(J)** between SDCBP2 and SLC7A11 expression in patients’ LUAD tissues. **(K, L)** OS analysis in TCGA-LUAD cohort. *P < 0.05; **P < 0.01; ***P < 0.001.

To investigate the role of SDCBP2 in ferroptosis, we measured reactive oxygen species (ROS) and glutathione (GSH) levels in lung adenocarcinoma cell lines (A549 and PC-9). The results demonstrated that SDCBP2 knockdown significantly increased intracellular ROS levels and significantly decreased GSH levels (**Figures 8A, B**), suggesting that downregulation of SDCBP2 can promote ferroptosis in LUAD cells. To explore the detailed association between SDCBP2 and ferroptosis, we analyzed the expression differences of ferroptosis regulator genes in high and low SDCBP2 expression groups in the TCGA-LUAD and GSE72094 cohorts. The results indicated that there were 35 ferroptosis regulator genes in the TCGA-LUAD cohort and 22 in the GSE72094 cohort with statistically significant expression differences (**Figures 8C, D**). Secondly, we analyzed the correlation between SDCBP2 and the expression of ferroptosis regulator genes in both cohorts (**Figure 8E**), and found that 7 genes in the TCGA-LUAD cohort and 9 genes in the GSE72094 cohort had a strong correlation with SDCBP2 (| r | > 0.3 and P < 0.05). Integrating the correlation and differential analysis results from the GSE72094 and TCGA-LUAD cohorts, a total of 5 key ferroptosis regulator genes were selected, namely AIFM2, AKR1C1, AKR1C2, AKR1C3, and SLC7A11 (**Figure 8F**). Among them, SLC7A11 had the highest correlation with the expression of SDCBP2 (R = 0.47, P < 0.001, **Figure 8G**). Subsequently, *In vitro* experiments revealed that SLC7A11 protein levels were markedly reduced in A549 and PC-9 cells transfected with SDCBP2 siRNA (**Figure 8H**). IHC staining further demonstrated that both SDCBP2 and SLC7A11 were significantly overexpressed in tumor tissues compared with adjacent normal tissues from a total of 15 LUAD patients (**Figure 8I**), and their expression exhibited a significant positive correlation (R = 0.522, P = 0.046, **Figure 8J**). Prognostic analysis in TCGA-LUAD cohort indicated that SDCBP2 and SLC7A11 functioned as synergistic prognostic markers in LUAD (**Figures 8K, L**). Collectively, these findings suggest that the expression of SDCBP2 is correlated with SLC7A11, and that downregulation of SDCBP2 can significantly promote ferroptosis in LUAD cells.

## 4 Discussion

In this study, we identified a novel ferroptosis-related prognostic biomarker SDCBP2 (syndecan-binding protein 2) by constructing a ferroptosis risk model in LUAD. Analysis of the TCGA pan-cancer dataset revealed that SDCBP2 is a prognostic risk gene only in LUAD. Further analysis of the TCGA-LUAD cohort found that SDCBP2 is highly expressed in LUAD and is associated with patient gender and tumor stage. Multivariable Cox regression analysis identified SDCBP2 expression, like pathological staging, as an independent prognostic factor for LUAD patients. Enrichment analysis of differentially expressed genes between high and low SDCBP2 expression groups in LUAD revealed that SDCBP2 may be closely related to biological processes such as the cell cycle, DNA replication, and cellular senescence. *In vitro* experiments showed that in LUAD, SDCBP2, acting as an oncogene, its downregulation inhibits the proliferation and migration of LUAD cells and induces G1 phase cell cycle arrest and apoptosis. Finally, we investigated the role of SDCBP2 in ferroptosis and found that SDCBP2 may affect ferroptosis in LUAD, ultimately influencing the prognosis of patients.

SDCBP2, also known as Syntenin-2, encodes a protein that can bind with high affinity to PIP2 (phosphatidylinositol 4,5-bisphosphate) through its PDZ domain and is involved in the regulation of cell division (17). PIP2, as a substrate for phospholipase C (PLC) and phosphatidylinositol-3 kinase (PI3K), participates in the regulation of the PTEN/PI3K/AKT signaling pathway and plays an important role in the proliferation, metabolism, and apoptosis of tumor cells (18). Studies have found that SDCBP2 expression is increased in patients with acute myeloid leukemia (AML), and downregulating SDCBP2 expression can inhibit the proliferation of AML cells and induce their differentiation (19). However, the role of SDCBP2 in solid tumors, including LUAD, has not yet been reported. Although studies have indicated that syndecan-binding protein 2-antisense RNA 1 (SDCBP2-AS1) acts as a tumor suppressor and can inhibit the proliferation and metastasis of gastric carcinoma (GC) cells, there is no correlation between SDCBP2 and SDCBP2-AS1 at the post-transcriptional level (20). In this study, we report for the first time the oncogenic role and prognostic value of SDCBP2 in LUAD and further validate its potential in regulating cell cycle and apoptosis through *in vitro* experiments. These results prove that SDCBP2 may become a new potential therapeutic target in LUAD.

Ferroptosis, as a potential therapeutic strategy for treating drug-resistant cancer types, is gaining increasing attention, particularly in lung cancer, where it is evident in addressing resistance to targeted therapies (21, 22). Our studies indicate that SDCBP2 may be closely associated with the KEAP1–NRF2 (also known as NFE2L2) – SLC7A11 pathway. NRF2 (Nuclear factor erythroid 2-related factor 2), as a master transcription factor of antioxidation, plays a key role in ferroptosis through regulating GSH metabolism, intracellular free iron content, mitochondrial function, lipid metabolism, etc. Specifically, NRF2 binds directly to the sequence of antioxidant response element (ARE) locating in SLC7A11 (solute carrier family 7 membrane 11) promoter region and then promotes the expression of SLC7A11, thereby increasing GSH synthesis and ultimately suppressing ferroptosis. And KEAP1 (Kelch ECH-associated protein 1) can promote the ubiquitination of NRF2, thereby targeting it for proteasomal degradation (8, 23–26). Furthermore, recent research has proposed a new ferroptosis inhibitor—AIFM2 (Apoptosis Inducing Factor Mitochondrion 2)/FSP1 (Ferroptosis Suppressor Protein 1), which captures lipid peroxides in a GPX4 (Glutathione Peroxidase 4)-independent manner (27, 28). In our study, SDCBP2 showed correlations with the expression of AIFM2, SLC7A11, and AKR1C family genes, suggesting that SDCBP2 may regulate ferroptosis through multiple pathways and is a potential ferroptosis regulator gene.

In summary, this study demonstrates through bioinformatics analysis and *in vitro* experiments that SDCBP2 is a novel ferroptosis-related prognostic biomarker and promotes the progression of LUAD by regulating by affecting G1-phase cell cycle arrest, cell apoptosis, and ferroptosis. However, there are some limitations to this study. Firstly, the mechanism by which SDCBP2 regulates the cell cycle and apoptosis in LUAD tumor cells still needs further investigation; we have not addressed the specific regulatory relationships between SDCBP2 and cell cycle and apoptosis-related molecular targets. Secondly, the impact of SDCBP2 on ferroptosis and its underlying mechanism remain to be further investigated. Our experiments did not provide direct evidence of changes in ferroptosis, as only limited measurements of ROS/GSH levels were conducted. Moreover, although we identified and validated the correlation between SDCBP2 expression and SLC7A11, this does not establish a definitive regulatory relationship between them. Lastly, the oncogenic role of SDCBP2 in LUAD still needs to be further validated in animal models and patient tissue samples.

## 5 Conclusion

Our study revealed that SDCBP2, as a novel ferroptosis-related independent prognostic biomarker, plays a critical role in LUAD development by affecting G1-phase cell cycle arrest, cell apoptosis, and ferroptosis, suggesting that SDCBP2 could be a novel therapeutic target in LUAD.

## Data Availability

The raw data supporting the conclusions of this article will be made available by the authors, without undue reservation.

## Supplementary material

Supplementary Figure 1 The associations between SDCBP2 expression and clinicopathological parameters.

## References

[1] Bray F Laversanne M Sung H Ferlay J Siegel RL Soerjomataram I . Global cancer statistics 2022: GLOBOCAN estimates of incidence and mortality worldwide for 36 cancers in 185 countries. CA: Cancer J Clin. (2024) 74:229–63. doi: 10.3322/caac.21834, PMID: 38572751

[2] Testa U Pelosi E Castelli G . Molecular charcterization of lung adenocarcinoma combining whole exome sequencing, copy number analysis and gene expression profiling. Expert Rev Mol Diagn. (2022) 22:77–100. doi: 10.1080/14737159.2022.2017774, PMID: 34894979

[3] Mogavero A Bironzo P Righi L Merlini A Benso F Novello S . Deciphering lung adenocarcinoma heterogeneity: an overview of pathological and clinical features of rare subtypes. Life (Basel Switzerland). (2023) 13. doi: 10.3390/life13061291, PMID: 37374074 PMC10302096

[4] Wu J Lin Z . Non-small cell lung cancer targeted therapy: drugs and mechanisms of drug resistance. Int J Mol Sci. (2022) 23. doi: 10.3390/ijms232315056, PMID: 36499382 PMC9738331

[5] Hanson S Dharan A Jinsha PV Pal S Nair BG Kar R . Paraptosis: a unique cell death mode for targeting cancer. Front Pharmacol. (2023) 14:1159409. doi: 10.3389/fphar.2023.1159409, PMID: 37397502 PMC10308048

[6] Ergün S Aslan S Demir D Kayaoğlu S Saydam M Keleş Y . Beyond death: unmasking the intricacies of apoptosis escape. Mol Diagn Ther. (2024) 28:403–23. doi: 10.1007/s40291-024-00718-w, PMID: 38890247 PMC11211167

[7] Stockwell BR Friedmann Angeli JP Bayir H Bush AI Conrad M Dixon SJ . Ferroptosis: A regulated cell death nexus linking metabolism, redox biology, and disease. Cell. (2017) 171:273–85. doi: 10.1016/j.cell.2017.09.021, PMID: 28985560 PMC5685180

[8] Wei X Li X Hu S Cheng J Cai R . Regulation of ferroptosis in lung adenocarcinoma. Int J Mol Sci. (2023) 24. doi: 10.3390/ijms241914614, PMID: 37834062 PMC10572737

[9] Stockwell BR . Ferroptosis turns 10: Emerging mechanisms, physiological functions, and therapeutic applications. Cell. (2022) 185:2401–21. doi: 10.1016/j.cell.2022.06.003, PMID: 35803244 PMC9273022

[10] Zalewska-Ziob M Adamek B Kasperczyk J Romuk E Hudziec E Chwalińska E . Activity of antioxidant enzymes in the tumor and adjacent noncancerous tissues of non-small-cell lung cancer. Oxid Med Cell Longev. (2019) 2019:2901840. doi: 10.1155/2019/2901840, PMID: 31781331 PMC6875225

[11] Lei G Zhuang L Gan B . Targeting ferroptosis as a vulnerability in cancer. Nat Rev Cancer. (2022) 22:381–96. doi: 10.1038/s41568-022-00459-0, PMID: 35338310 PMC10243716

[12] Zhang C Liu X Jin S Chen Y Guo R . Ferroptosis in cancer therapy: a novel approach to reversing drug resistance. Mol Cancer. (2022) 21:47. doi: 10.1186/s12943-022-01530-y, PMID: 35151318 PMC8840702

[13] Chen X Kang R Kroemer G Tang D . Broadening horizons: the role of ferroptosis in cancer. Nat Rev Clin Oncol. (2021) 18:280–96. doi: 10.1038/s41571-020-00462-0, PMID: 33514910

[14] Jiang X Stockwell BR Conrad M . Ferroptosis: mechanisms, biology and role in disease. Nat Rev Mol Cell Biol. (2021) 22:266–82. doi: 10.1038/s41580-020-00324-8, PMID: 33495651 PMC8142022

[15] Ritchie ME Phipson B Wu D Hu Y Law CW Shi W . limma powers differential expression analyses for RNA-sequencing and microarray studies. Nucleic Acids Res. (2015) 43:e47. doi: 10.1093/nar/gkv007, PMID: 25605792 PMC4402510

[16] Subramanian A Tamayo P Mootha VK Mukherjee S Ebert BL Gillette MA . Gene set enrichment analysis: a knowledge-based approach for interpreting genome-wide expression profiles. Proc Natl Acad Sci United States America. (2005) 102:15545–50. doi: 10.1073/pnas.0506580102, PMID: 16199517 PMC1239896

[17] Mortier E Wuytens G Leenaerts I Hannes F Heung MY Degeest G . Nuclear speckles and nucleoli targeting by PIP2-PDZ domain interactions. EMBO J. (2005) 24:2556–65. doi: 10.1038/sj.emboj.7600722, PMID: 15961997 PMC1176451

[18] Liu A Zhu Y Chen W Merlino G Yu Y . PTEN dual lipid- and protein-phosphatase function in tumor progression. Cancers. (2022) 14. doi: 10.3390/cancers14153666, PMID: 35954330 PMC9367293

[19] Du Y Li LL Chen F Du Y . Targeting SDCBP2 in acute myeloid leukemia. Cell Signalling. (2023) 112:110889. doi: 10.1016/j.cellsig.2023.110889, PMID: 37714445

[20] Han J Nie M Chen C Cheng X Guo T Huangfu L . SDCBP-AS1 destabilizes β-catenin by regulating ubiquitination and SUMOylation of hnRNP K to suppress gastric tumorigenicity and metastasis. Cancer Commun (London England). (2022) 42:1141–61. doi: 10.1002/cac2.12367, PMID: 36209503 PMC9648392

[21] Yapici FI Bebber CM von Karstedt S . A guide to ferroptosis in cancer. Mol Oncol. (2024) 18:1378–96. doi: 10.1002/1878-0261.13649, PMID: 38590214 PMC11161738

[22] Kang N Son S Min S Hong H Kim C An J . Stimuli-responsive ferroptosis for cancer therapy. Chem Soc Rev. (2023) 52:3955–72. doi: 10.1039/D3CS00001J, PMID: 37218295

[23] Anandhan A Dodson M Shakya A Chen J Liu P Wei Y . NRF2 controls iron homeostasis and ferroptosis through HERC2 and VAMP8. Sci Adv. (2023) 9:eade9585. doi: 10.1126/sciadv.ade9585, PMID: 36724221 PMC9891695

[24] Song X Long D . Nrf2 and ferroptosis: A new research direction for neurodegenerative diseases. Front Neurosci. (2020) 14:267. doi: 10.3389/fnins.2020.00267, PMID: 32372896 PMC7186402

[25] Rojo de la Vega M Chapman E Zhang DD . NRF2 and the hallmarks of cancer. Cancer Cell. (2018) 34:21–43. doi: 10.1016/j.ccell.2018.03.022, PMID: 29731393 PMC6039250

[26] Mills EL Ryan DG Prag HA Dikovskaya D Menon D Zaslona Z . Itaconate is an anti-inflammatory metabolite that activates Nrf2 via alkylation of KEAP1. Nature. (2018) 556:113–7. doi: 10.1038/nature25986, PMID: 29590092 PMC6047741

[27] Bersuker K Hendricks JM Li Z Magtanong L Ford B Tang PH . The CoQ oxidoreductase FSP1 acts parallel to GPX4 to inhibit ferroptosis. Nature. (2019) 575:688–92. doi: 10.1038/s41586-019-1705-2, PMID: 31634900 PMC6883167

[28] Doll S Freitas FP Shah R Aldrovandi M da Silva MC Ingold I . FSP1 is a glutathione-independent ferroptosis suppressor. Nature. (2019) 575:693–8. doi: 10.1038/s41586-019-1707-0, PMID: 31634899

