# Supplementary material for: SDCBP2 promotes tumor progression and is a novel ferroptosis-related prognostic biomarker in lung adenocarcinoma
Source: Front Immunol. 2025 Dec 2;16:1692308. doi: 10.3389/fimmu.2025.1692308 (PMC12705533; doi:10.3389/fimmu.2025.1692308)
Supplement: Supplementary file 1 [file Table1.docx]

**Supplementary Table 1.** SiRNAs sequences used in SDCBP2 knockdown experiment.

| Name | Sense or antisense | siRNA sequence |
| --- | --- | --- |
| Negative control siRNA | sense sequence | 5ʹ-UUCUCCGAAGGUGUCACGUTT-3ʹ |
|  | antisense sequence | 5ʹ-ACGUGACACGUUCGGAGAATT-3ʹ |
| siSDCBP2 siRNA-1 | sense sequence | 5ʹ-GCAGAAUGUUAUCGGGCUGTT-3ʹ |
|  | antisense sequence | 5ʹ-CAGCCCGAUAACAUUCUGCTT-3ʹ |
| siSDCBP2 siRNA-2 | sense sequence | 5ʹ-GAAGAUUGUCUCUCUGGUCTT-3ʹ |
|  | antisense sequence | 5ʹ-GACCAGAGAGACAAUCUUCTT-3ʹ |
