# Supplementary material for: SDCBP2 promotes tumor progression and is a novel ferroptosis-related prognostic biomarker in lung adenocarcinoma
Source: Front Immunol. 2025 Dec 2;16:1692308. doi: 10.3389/fimmu.2025.1692308 (PMC12705533; doi:10.3389/fimmu.2025.1692308)
Supplement: Supplementary file 2 [file Table2.docx]

**Supplementary Table 2.** Primer sequences for PCR.

| Primer | Sequence |
| --- | --- |
| H-SDCBP2-S | 5ʹ-TGACAGTACAGCGGTCTCGG-3ʹ |
| H-SDCBP2-A | 5ʹ-CCTTGCACAGGTGGATCTCG-3ʹ |
| H-ACTIN-S | 5ʹ-CACCCAGCACAATGAAGATCAAGAT-3ʹ |
| H-ACTIN-A | 5ʹ-CCAGTTTTTAAATCCTGAGTCAAGC-3ʹ |
