# Supplementary figures and images for: SDCBP2 promotes tumor progression and is a novel ferroptosis-related prognostic biomarker in lung adenocarcinoma
Source: Front Immunol. 2025 Dec 2;16:1692308. doi: 10.3389/fimmu.2025.1692308 (PMC12705533; doi:10.3389/fimmu.2025.1692308)

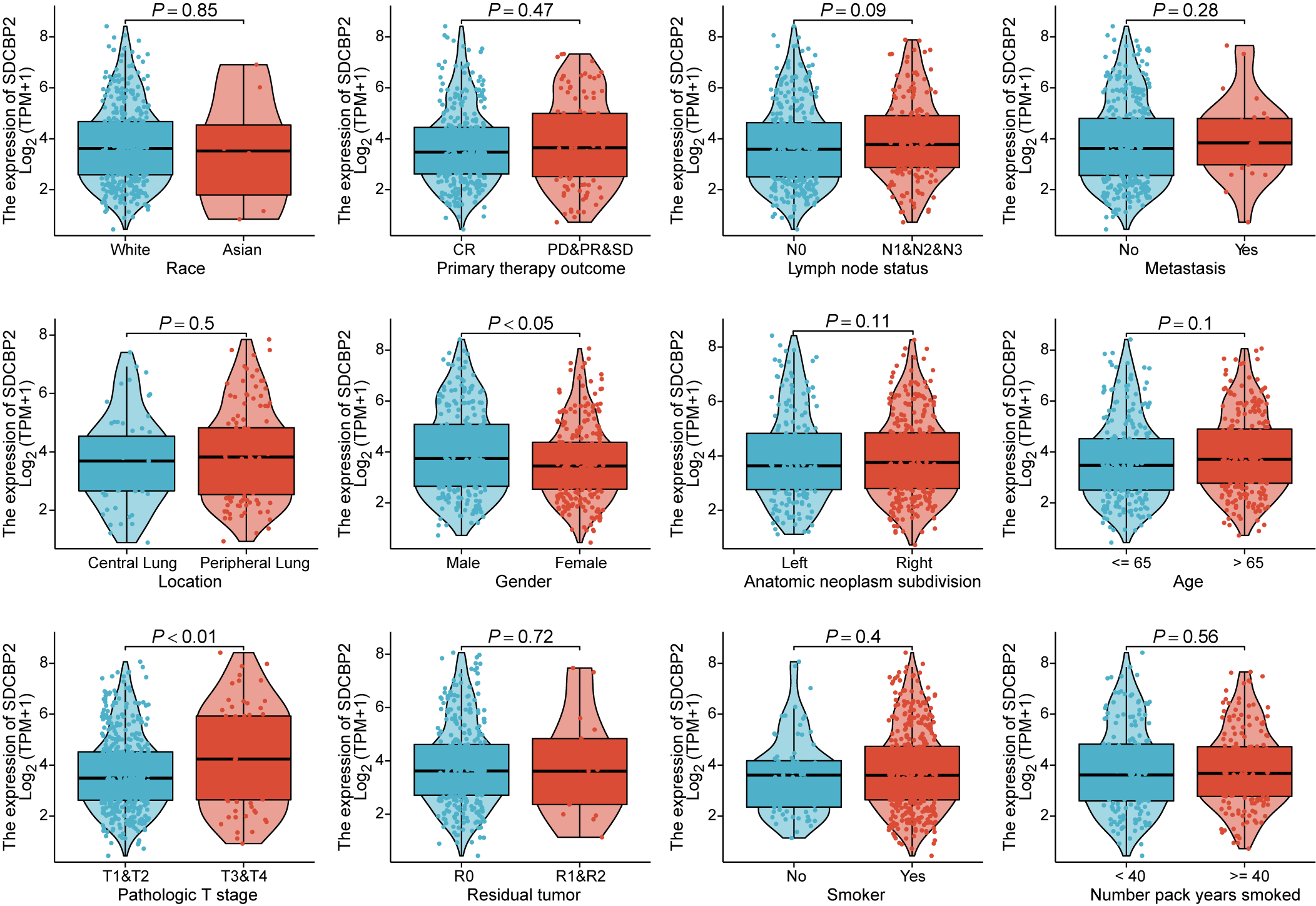

Supplement: Supplementary Figure 1 — The associations between SDCBP2 expression and clinicopathological parameters. [file Image1.tif]
